# The Hessian Blob Algorithm: Precise Particle Detection in Atomic Force Microscopy Imagery

**DOI:** 10.1038/s41598-018-19379-x

**Published:** 2018-01-17

**Authors:** Brendan P. Marsh, Nagaraju Chada, Raghavendar Reddy Sanganna Gari, Krishna P. Sigdel, Gavin M. King

**Affiliations:** 1 Department of Physics and Astronomy, University of Missouri, Columbia, Missouri 65211 United States of America; 2 Department of Biochemistry, University of Missouri, Columbia, Missouri 65211 United States of America; 3 Present Address: Department of Applied Mathematics and Theoretical Physics, University of Cambridge, Cambridge, CB3 OWA United Kingdom; 4 Present Address: School of Medicine, University of Virginia, Charlottesville, Virginia 22908 United States of America

## Abstract

Imaging by atomic force microscopy (AFM) offers high-resolution descriptions of many biological systems; however, regardless of resolution, conclusions drawn from AFM images are only as robust as the analysis leading to those conclusions. Vital to the analysis of biomolecules in AFM imagery is the initial detection of individual particles from large-scale images. Threshold and watershed algorithms are conventional for automatic particle detection but demand manual image preprocessing and produce particle boundaries which deform as a function of user-defined parameters, producing imprecise results subject to bias. Here, we introduce the Hessian blob to address these shortcomings. Combining a scale-space framework with measures of local image curvature, the Hessian blob formally defines particle centers and their boundaries, both to subpixel precision. Resulting particle boundaries are independent of user defined parameters, with no image preprocessing required. We demonstrate through direct comparison that the Hessian blob algorithm more accurately detects biomolecules than conventional AFM particle detection techniques. Furthermore, the algorithm proves largely insensitive to common imaging artifacts and noise, delivering a stable framework for particle analysis in AFM.

## Introduction

Atomic force microscopy (AFM) is a key tool in single molecule biophysics with the capability to image diverse biological systems with sub-nanometer lateral resolution^1,2^. Imaging assays via AFM are advancing in many directions, such as high-speed imaging for direct observation of dynamic molecular behavior^3^, allowing AFM to investigate complex biological systems. With these advances comes the demand for precise analysis methods making full use of high resolution data in an unbiased and reliable manner^4^.

AFM image analysis often starts with large-scale images containing tens to hundreds of individual biomolecules, with the goal of detecting, localizing, and parameterizing individual biomolecules. For simplicity, this procedure is often conducted manually. Yet, there are clear limitations to manual particle detection – the most pressing are that the process is time consuming and subject to human bias. As a result, different individuals may detect particles and delineate their boundaries differently. To minimize human bias and provide quantitative rules for particle detection, an automated detection algorithm is needed.

Standard automatic particle detection algorithms, such as the threshold and watershed algorithms found in the Gwyddion^5^ analysis software for scanning probe microscope images, are imprecise and poorly suited for precision single molecule studies. Threshold algorithms set a global height threshold to mask particles and thus require a flat background level to be effective, demanding background subtraction as a preprocessing step. Particle boundaries demonstrate direct dependence on the height threshold parameter, and are only well-founded under assumptions of flat, constant background levels. Watershed algorithms^6,7^ treat the image as a topographical map, fully segmenting the image into regions based on the flow of water into basins. By inverting an AFM image, biomolecule protrusions are converted into basins detectable by classical watershed algorithms. However, such algorithms are not well suited when the precise biomolecule boundary is desired, as watershed basins may expand beyond the extent of particles and always separate all peaks (local maxima) in the image. Watershed algorithms are therefore ineffective when studying biomolecules displaying multiple peaks, such as multimeric proteins. Moreover, this sensitivity to peaks includes peaks arising from single-pixel noise, which must be diminished by appropriate smoothing as a preprocessing step. Thus conventional particle detection algorithms, while popular, produce particles which depend on subjective image preprocessing and algorithm parameters, which, even when set appropriately, produce poorly founded boundaries.

Here, we address ill-founded boundaries and parameter dependence in particle detection algorithms for AFM. We primarily consider the concept of scale in image structures^8,9^ from the field of computer vision. Real world objects and image features alike are only well-defined over a limited range of scales - leaves may be described well on the scale of centimeters, forests on the scale of kilometers. Just as it is ineffective to describe a forest centimeter by centimeter, nor is it effective to analyze hundred-pixel features using single pixel characteristics. Features in AFM images range from small membrane protein protrusions only ~1 nm wide^10^ (captured by only a few pixels) to large aggregates and lattice structures^2,11,12^ (hundreds of pixels). Small features indeed require single pixel analysis, but larger scale features are better understood by looking at aggregate pixel behavior at some scale. Determining optimal scales is common in image analysis, appearing in contexts ranging from Canny edge detection^13^ to adaptive filtering^14^. Thus, we must first determine how to deduce an appropriate scale for a biomolecule within an AFM image and then specify how to analyze the biomolecule at that scale.

Addressing both challenges, scale-space theory^9^ links the concept of scale with Gaussian smoothing. Informally, the scale-space representation of an image at some scale, say ten pixels, is formed by smoothing the image such that features smaller than ten pixels are blurred out while larger features remain identifiable. The full scale-space representation is the collection of all smoothed images, where the scale (relating to the amount of smoothing) increases from zero (no smoothing) to infinity (completely smooth image). For a discrete AFM image, the scale-space representation takes the form of a three-dimensional stack of images, where the smoothing level increases at higher layers of the stack. This makes working with a biomolecule at any scale easy, we simply view the particle at some layer (at some level of smoothing) in the image stack.

Determining the scale of a biomolecule is called scale-selection. This and more may be accomplished via scale-space interest point detectors. These functions produce local extrema centered on various kinds of low-level image features such as blobs, edges, or junctions – providing both the spatial location of these features as well as their scale^15,16^. To first order, biomolecules may be regarded as ‘blobs’, a common term in the field of computer vision referring to roughly homogenous regions of pixels, and are detectable by the class of interest point detectors known as blob detectors. Blob detection has been used extensively in contexts ranging from medical imagery^17–19^ to infrared military data^20^ and is well suited for the detection of molecule center points in AFM images.

Blob detection may provide the center points and scales of biomolecules in AFM images, but it remains to determine a well-founded boundary around a biomolecule. The boundary and resulting shape of a biomolecule is critically important for measures of particle volume and area, which are commonly used metrics in AFM studies^5,10^. Algorithms incorporating the scale-space representation have proven powerful, especially when combined with measures of the image curvature, often measured via the Hessian matrix. The principal curvature-based region algorithm^21^ uses watersheds of a maximum curvature image computed from the scale-space representation, providing stable regions of interest in an image. Developed to detect kidney glomeruli in magnetic resonance imaging, the Hessian-based difference of Gaussian method^22^ delineates boundaries based on convexity of the difference of Gaussian scale-space representation. Various other methods have been proposed^23–25^. Here, we extend Hessian-based curvature in scale-space to delineate particle boundaries, based on an intuitive definition which yields robust subpixel precision.

In particular, we present the Hessian blob, offering a definition of a particle founded in scale-space blob detection and Gaussian curvature, with a straightforward extension to subpixel precise particle boundaries and center points. We demonstrate that the Hessian blob algorithm consistently detects biomolecules in AFM images and defines smooth boundaries with high precision. Moreover, particle shape dependence on user-defined algorithm parameters, as seen for the standard AFM particle detections algorithms, is eliminated. We show by direct comparison that the Hessian blob algorithm is well equipped to handle the challenges posed in AFM images, more so than conventional particle detection algorithms.

## Biomolecule Center Point Detection via Scale-Space Blob Detection

Biomolecules in AFM images may be regarded at a low level as image “blobs”. The blob is a well-established concept in the field of computer vision^9,15^ and may be described as a set of connected pixels which are *roughly* homogenous by some measure. This roughness captures the idea that blobs should not depend heavily on single pixel values, but on behavior defined at some characteristic scale, which is usually not known a priori. The scale-space representation, covering a range of scales, thus provides an appropriate framework for blob detection. A brief introduction to scale-space blob detection is given here to provide a working understanding with further details in the Supplemental Information.

Consider an AFM image as a function $I(x,y)$ giving the pixel intensity (which is usually proportional to height) at any point in the image. We regard the discrete scale-space representation^8,9^ *L*(*x*, *y*; *t*) as a family of images derived from *I*, with each image indexed by a scale *t*. For any positive scale *t*_0_, the scale-space representation *L*(*x*, *y*; *t*_0_) is an image derived from *I*(*x*, *y*) through discrete Gaussian smoothing^26–28^. The full scale-space representation *L*(*x*, *y*; *t*) is then a stack of smoothed images for all relevant scales *t*, with increasing scale (increasing smoothing) at higher levels of the stack. While the scale may assume any positive value, a natural lower bound arises from the pixel spacing of the image (generally around 1 nm in AFM images) and an upper bound is suggested by the total size of the image (typically microns), with the scale sampled in geometric steps in between.

Blob detection occurs within the three-dimensional scale-space domain, with the two familiar spatial coordinates *x* and *y* along with the introduced scale coordinate *t*. Differential blob detectors^15^ may then be constructed as functions in scale-space which attain (three dimensional) local extrema on blob centers at their corresponding scale. The challenge of detecting blobs is thus reduced to detecting local extrema of blob detector functions. Differential blob detectors are composed from derivatives of the scale-space representation, written as ${L}_{{x}^{\alpha }{y}^{\beta }}={\partial }_{{x}^{\alpha }{y}^{\beta }}\,L(x,y;t)$ where ${\partial }_{{x}^{\alpha }{y}^{\beta }}=({\partial }^{\alpha }/\partial {x}^{\alpha })\,({\partial }^{\beta }/\partial {y}^{\beta })$, and normalized according to their scale to preserve the magnitude of derivatives after smoothing. The Hessian blob algorithm presented here uses two of the simplest blob detectors, the normalized Laplacian of Gaussian ${\nabla }_{norm}^{2}L$ and determinant of the Hessian ${\rm{\det }}\,{ {\mathcal H} }_{norm}L$. Both are rotationally invariant expressions derived from the Hessian matrix which may be shown, by direct analysis on blob models, to recover the position and scale of image structures^29^.

1 $${{\rm{\nabla }}}_{norm}^{2}L={\rm{t}}{\rm{r}}(\begin{array}{cc}t{{\rm{\partial }}}_{xx} & t{{\rm{\partial }}}_{xy}\\ t{{\rm{\partial }}}_{yx} & t{{\rm{\partial }}}_{yy}\end{array})L=t({L}_{xx}+{L}_{yy})\,\quad \,\,\,\,\,det\,{{\mathscr{H}}}_{norm}L=det\,(\begin{array}{cc}t{{\rm{\partial }}}_{xx} & t{{\rm{\partial }}}_{xy}\\ t{{\rm{\partial }}}_{yx} & t{{\rm{\partial }}}_{yy}\end{array})={t}^{2}({L}_{xx}{L}_{yy}-{L}_{xy}^{2})$$

In terms of their general behavior, both ${\nabla }_{norm}^{2}L$ and ${\rm{\det }}\,{ {\mathcal H} }_{norm}L$ produce extrema located on blob centers with the scale *t* and approximate radius of the blob *r* satisfying $r=\sqrt{2t}$, thus in many cases the two blob detectors produce similar results. However, only ${\nabla }_{norm}^{2}L$ preserves the sign of the blob, producing minima for bright blobs (greater than the background level, like a mound) and maxima for dark blobs (less than the background level, like a crater). In contrast, ${\rm{\det }}\,{ {\mathcal H} }_{norm}L$ is maximal for both bright and dark blobs. Despite their often similar detection abilities, ${\rm{\det }}\,{ {\mathcal H} }_{norm}L$ proves consistent under affine image transformations and demonstrates stronger repeatability properties than most other blob detectors^29,30^. Thus, ${\rm{\det }}\,{ {\mathcal H} }_{norm}L$ remains one of the most robust blob detectors and is used to construct Hessian blobs, whereas ${\nabla }_{norm}^{2}L$ is used solely to recover sign information.

Blob detectors may further be used to analyze the image scale structure at any point. For a fixed spatial point $({x}_{0},{y}_{0})$ in an image, the response ${\nabla }_{norm}^{2}L({x}_{0},{y}_{0};t)$ as a function of the scale *t* is known as the scale-space signature. Scales which produce local extrema of the scale-space signature may be used to generate hypotheses about natural scales of the image structure at that point^15^.

**Figure 1:**
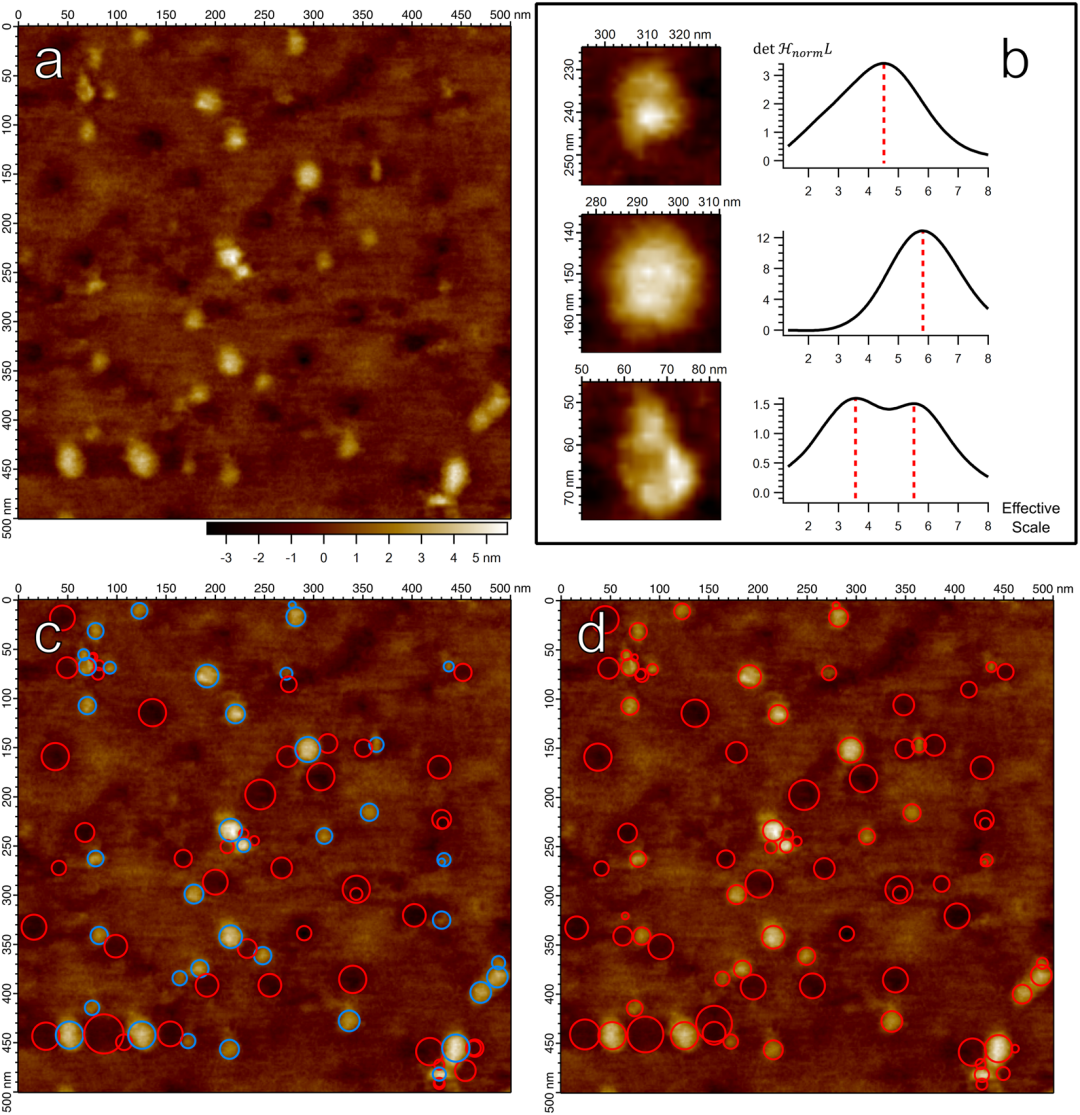
(**a**) AFM image of membrane protein SecYEG embedded in a lipid bilayer supported on a glass substrate^11^. (**b**) Particles from (**a**) with increased color contrast, and their corresponding scale-space signature to their right. The response of ${\rm{\det }}\,{ {\mathcal H} }_{norm}L$ scaled by a factor of 1 × 10^19^ was calculated at the central pixel in each particle and plotted as a function of the effective scale^44^ approximated as ${\mathrm{log}}_{2}(1+t)$. Top Row: A smaller particle and the corresponding scale extracted from the scale-space signature, marked by red dashed line. Middle Row: A larger particle, with a detected scale greater than the top particle. Bottom Row: A complex particle with substructure components. Two scales are identified, one corresponding to a smaller substructure component and one for the particle as a whole. (**c**) The 90 strongest scale-space extrema of $\,{\nabla }_{norm}^{2}L$, represented as circles centered on the spatial coordinates of the scale-space extremum with radius $\sqrt{2t}$. As in the style used by Lindeberg^29^, red circles represent maxima (dark blobs), blue represent minima (bright blobs). (**d**) The 90 strongest scale-space maxima of ${\rm{\det }}\,{ {\mathcal H} }_{norm}L$, also as circles with radius $\sqrt{2t}$. Both positive and negative blobs correspond to maxima of ${\rm{\det }}\,{ {\mathcal H} }_{norm}L$.

Figure 1 demonstrates the success of both ${\nabla }_{norm}^{2}L$ and ${\rm{\det }}\,{ {\mathcal H} }_{norm}L$ in identifying individual membrane proteins as well as their scale, with identified particles and scales matching well between the two blob detectors. However, the approximation of the blob boundary as a circle with radius related to the scale is crude; a more refined blob boundary is needed for high-precision measurements.

An important point made evident in Fig. 1b is that there may exist multiple maximal points in the scale-space signature. This generally suggests emergent structure at different scales – blobs within larger blobs. The first two particles (Fig. 1b top row and middle row) demonstrate single maxima in their scale-space signature, and correspondingly both of these blobs do not show any obvious finer structure. However, the final particle (Fig. 1b bottom row) demonstrates more complicated substructure, with at least two separate domains combined to form one larger unit. Likewise, the first maximum attained in the scale-space signature identifies the scale of a smaller domain while the second maximum identifies the scale corresponding to the particle as a whole. The scale-space signature thus presents itself as an indicator of emergent substructure within a biomolecule, with future implications in the study of oligomers and protein substructure.

## Biomolecule Boundary Detection via Gaussian Curvature

Precise delineation of particle boundaries is needed for high precision calculation of biomolecule volumes and areas. Moreover, a well-founded boundary definition is needed to provide a criterion for splitting or joining close particles, instead of leaving this choice to user interpretation or parameter settings. While blob detectors can robustly identify the central location and scale of biomolecules in AFM imagery, they cannot robustly localize boundaries.

A natural idea is to use image edges for particle boundaries. Various formulations exist, but an edge point is often defined geometrically as a point in an image at which the gradient magnitude assumes a local maximum in the gradient direction^16,31^. Many edge detection schemes have been put forth, with Canny edge detection^13^ remaining particularly popular and effective. Moreover, differential edges^16^ proposed by Lindeberg extend edges to scale-space, defined similarly as points in scale-space at which the gradient magnitude is locally maximal in the gradient direction and is maximal by some measure of edge saliency in the scale direction. Unfortunately, edges alone provide no guarantee of drawing closed contours around blobs. It is vitally important for particles to be well localized, thus edges in general cannot provide a complete description of blob boundaries.

The Hessian blob is based on a simple idea: blobs are geometrically different than saddle surfaces. At any point on a saddle surface there is opposing curvature: the surface curves upward in some directions and downward in others, producing the characteristic saddle shape. On the other hand, at any point on a bright blob the surface curves down in all directions, producing a convex surface. Thus, the scale-space representation of a blob should be locally convex at all points, not saddle-like. Convexity may be measured by the Gaussian curvature^32^ *K*, the product of the two principal curvatures $K={\kappa }_{1}{\kappa }_{2}$. The sign of *K* yields insightful information about the local structure of a surface – points on a surface which are locally convex have positive Gaussian curvature while saddle-like points have negative Gaussian curvature. Moreover, the Gaussian curvature may be computed directly from the scale-space representation to consider curvature at any scale. We then define the boundary (lateral extent) of a blob, at some scale, as the connected set of pixels for which the Gaussian curvature of the scale-space representation remains positive.

Regarding the scale-space representation for any fixed scale as a continuously differentiable function of two variables, the Gaussian curvature *K* and the determinant of the Hessian ${\rm{\det }}\, {\mathcal H} L$ are intimately related^32^.

2 $$K=\frac{\det  {\mathcal H} L}{{(1+{L}_{x}^{2}+{L}_{y}^{2})}^{2}}$$

We see the Gaussian curvature is the ratio of the determinant of the Hessian and an expression which is always positive. Sign information is therefore conserved, allowing us to equivalently define blob regions as sets of connected pixels for which ${\rm{\det }}\,{ {\mathcal H} }_{norm}L$ remains positive. Blob boundaries are thus formed by zero-crossings of ${\rm{\det }}\,{ {\mathcal H} }_{norm}L$. Given continuity, one cannot move from a positive region to a negative region without traversing a zero-crossing, implying that these boundaries form closed contours unlike traditional image edges.

**Figure 2:**
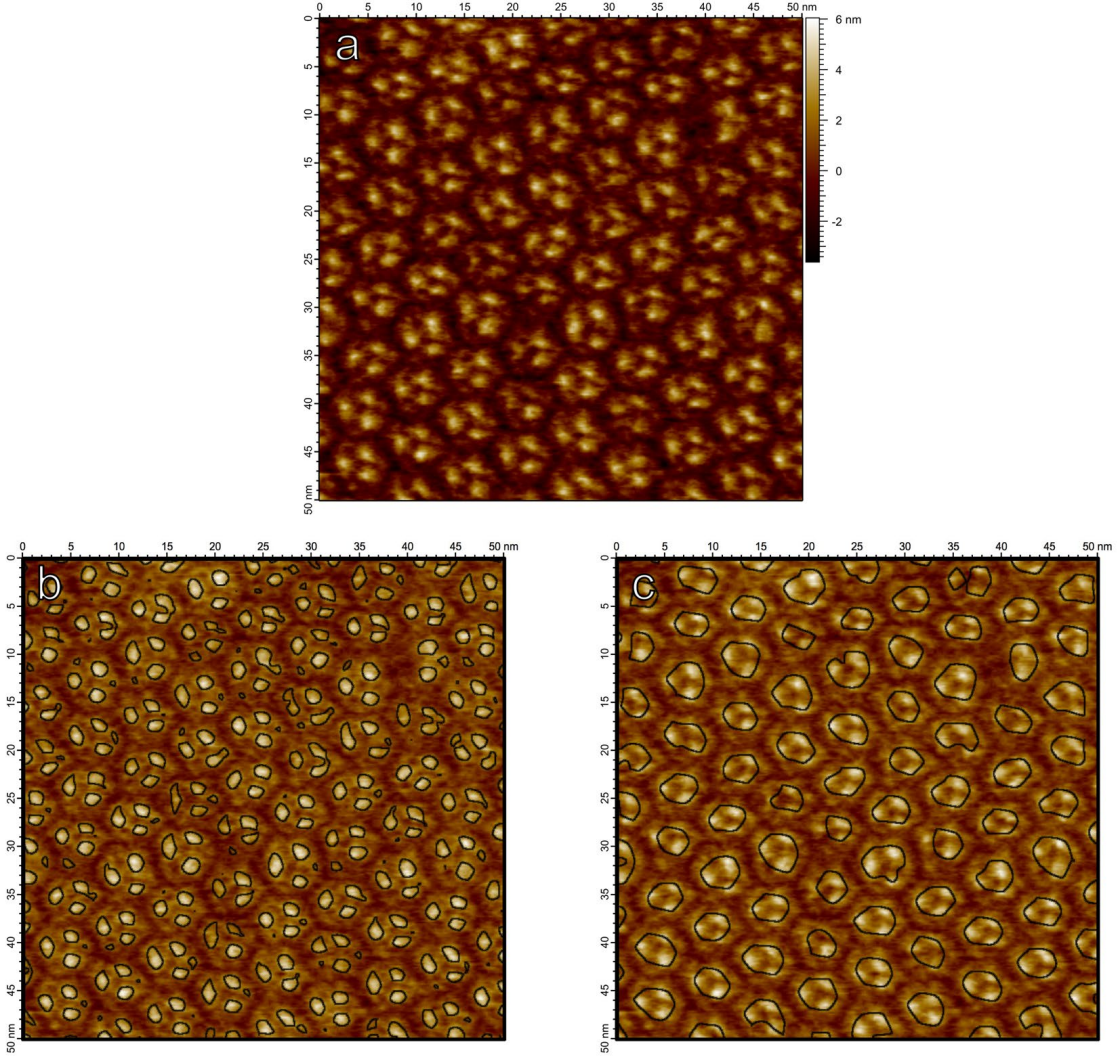
(**a**) AFM image of bacteriorhodopsin trimers in their natural lattice formation on a mica substrate. (**b**) Zero-crossings of Gaussian curvature computed on the scale-space representation of (**a**) at scale $t={(0.6{\rm{nm}})}^{2}$, overlaid on a brightened version of the image. At this finer scale, individual bacteriorhodopsin monomers are identified. (**c**) Zero-crossings of Gaussian curvature of the scale-space representation of (**a**) at the coarser scale $t=\,{(1.2{\rm{nm}})}^{2}$. At larger scales, the trimer structure becomes prevalent.

Figure 2 demonstrates how ${\rm{\det }}\,{ {\mathcal H} }_{norm}L$ can identify blob boundaries defined on different scales. Bacteriorhodopsin forms a highly organized lattice structure in the lipid bilayer composed of hexagonal trimer units, the trimer units themselves composed of three monomers. In Fig. 2b, zero-crossings of ${\rm{\det }}\,{ {\mathcal H} }_{norm}L$ associated with bright blobs are computed at a finer scale in scale-space, at which the smaller monomer units of bacteriorhodopsin are evident. Figure 2c again demonstrates zero-crossings of ${\rm{\det }}\,{ {\mathcal H} }_{norm}L$ but at a coarser scale in scale-space, at which the trimer structure as a whole is emergent. The zero-crossings of ${\rm{\det }}\,{ {\mathcal H} }_{norm}L$ in scale-space thus provide useful bounds for image structures which appear at different scales, even in the case of nested structures demonstrated here by bacteriorhodopsin.

## Complete Biomolecule Detection as Hessian Blobs

The Hessian blob, with subpixel precision, identifies emergent image structures through their center point position, their scale, and their boundary. The Hessian blob is motivated by the two simple observations demonstrated in the previous sections. First, scale-space blob detection was shown to be an effective method for determining the scale and center point of blobs. Second, stemming from the argument that blobs do not look like saddle points, we demonstrated that Gaussian curvature in scale-space is an effective method of delineating blob boundaries at any scale. Putting the pieces together, to define a Hessian blob we first detect the blob and its scale via scale-space blob detection, then delineate the boundary using Gaussian curvature at the detected scale.

**Figure 3:**
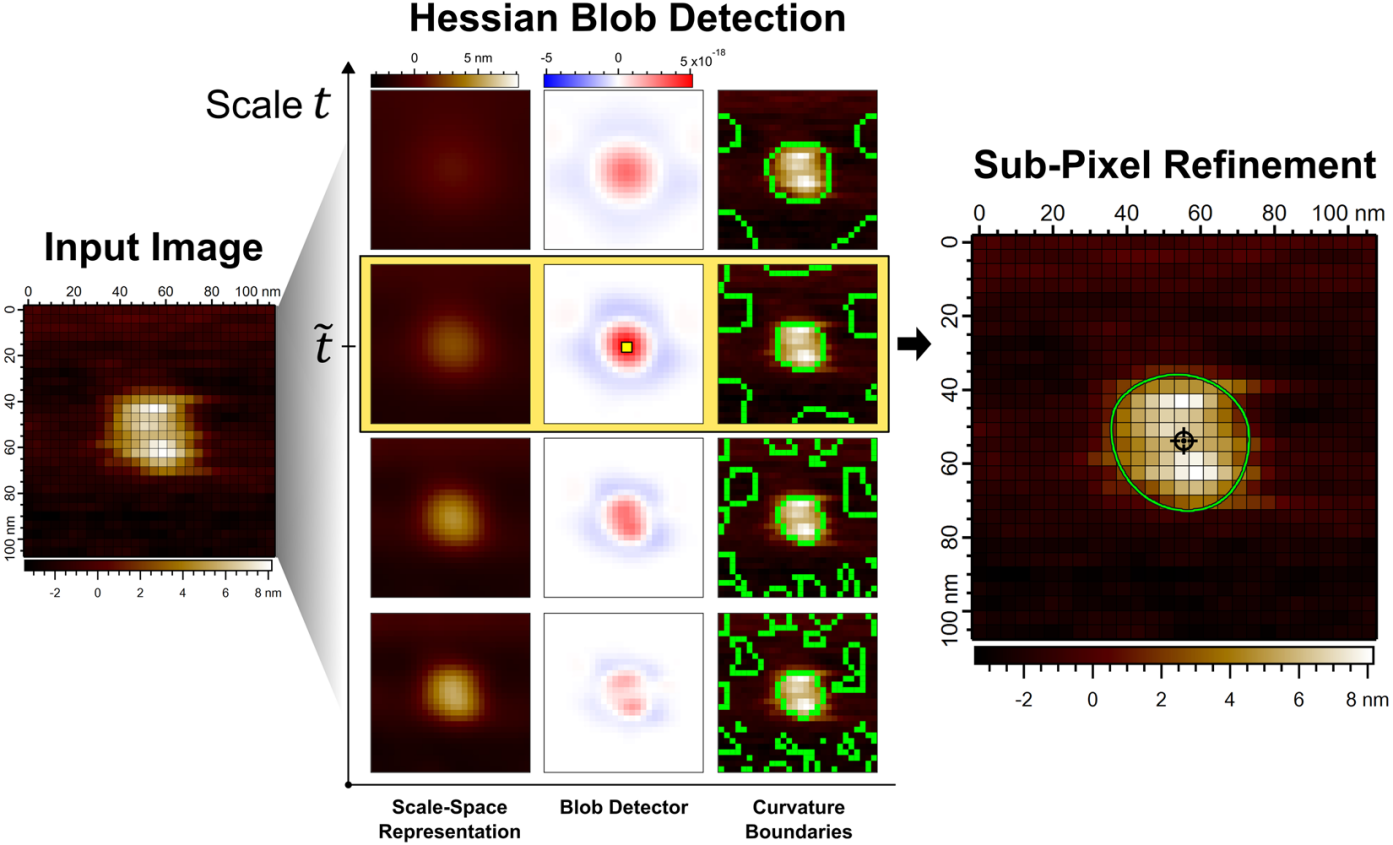
Visual demonstration of the Hessian blob algorithm applied to an image of a translocase complex (SecYEG/SecA)^41^ with lateral pixel resolution 3.9 nm. From an input image, the scale-space representation $L(x,y;t)$ is computed by discrete Gaussian smoothing, producing a stack of images indexed by the scale *t*, shown on the vertical axis. Differentiation and normalization yields $\det \,{ {\mathcal H} }_{norm}L(x,y;t)$, the blob detector, also a stack of images indexed by the scale *t*. Maximization of ${\rm{\det }}\,{ {\mathcal H} }_{norm}L$ in space and scale yields a scale-space maximum, highlighted by the yellow pixel, giving the blob center point in scale-space and selecting scale $t=\tilde{t}$. as the scale of the blob. In the third column, zero-crossings of the Gaussian curvature *K* are illustrated at different scales by green lines overlaid on the original image. Zero-crossings at scale $t=\tilde{t}$, the scale of the detected blob, are used to define the Hessian blob boundary. Sub-pixel refinements to the blob center point and boundary are finally computed, with the sub-pixel precise center point marked by a crosshair and boundaries computed to twenty times the lateral pixel resolution (0.20 nm).

Formally, the Hessian blob defines blob centers as scale-space maxima of ${\rm{\det }}\,{ {\mathcal H} }_{norm}L$, the sign of the blob being recovered by ${\nabla }^{2}L$. The spatial extent of the Hessian blob is then the region of positive ${\rm{\det }}\,{ {\mathcal H} }_{norm}L$ around the scale-space maxima, at the scale fixed by the scale-space maximum. Algorithm implementation details and the complete algorithm workflow are provided in the Supplemental Information. An algorithm overview is presented in Fig. 3.

Although AFM can exhibit resolution of biological macromolecules at <1 nm^1^, atomic structure still exists well below the pixel resolution. Hessian blob center points and boundaries may be computed to subpixel precision to estimate structure on a finer scale than the pixel spacing. A method for subpixel localization of scale-space interest points via the second order Taylor approximation was proposed by Brown and Lowe and is implemented in the SIFT algorithm^33,34^. Here, we apply this method directly to the blob detector $\det \,{ {\mathcal H} }_{norm}L(x,y;t)$, providing a second order approximation of the subpixel biomolecule center. Subpixel localization of the boundary may be approximated via interpolation of ${\rm{\det }}\,{ {\mathcal H} }_{norm}L$, with zero-crossings then identified in the same manner, as regions of positive $\det \,{ {\mathcal H} }_{norm}L$. In general, the accuracy of the interpolated boundary will depend on the method of interpolation and the form of the underlying function being approximated. Making no assumptions about the true topography of the sample, we do not identify any optimal subpixel resolution but choose the subpixel resolution as desired. Numerous interpolation methods exist^35,36^, though we note bicubic interpolation may be chosen to preserve continuous derivatives of the blob boundary, whereas bilinear interpolation may be chosen for fast computation.

Hessian blobs may overlap when projected out of scale-space back to the image domain. Here, in the case of two overlapping blobs, we only consider the blob with a stronger blob response – the value of ${\rm{\det }}\,{ {\mathcal H} }_{norm}L$ at the blob center. The weaker blob is then discarded. This algorithm is by no means the only solution; in fact, consideration of overlapping and nested blob structure is vital in the analysis of oligomeric state of proteins (composed of multiple units) and in the analysis of protein substructure.

**Figure 4:**
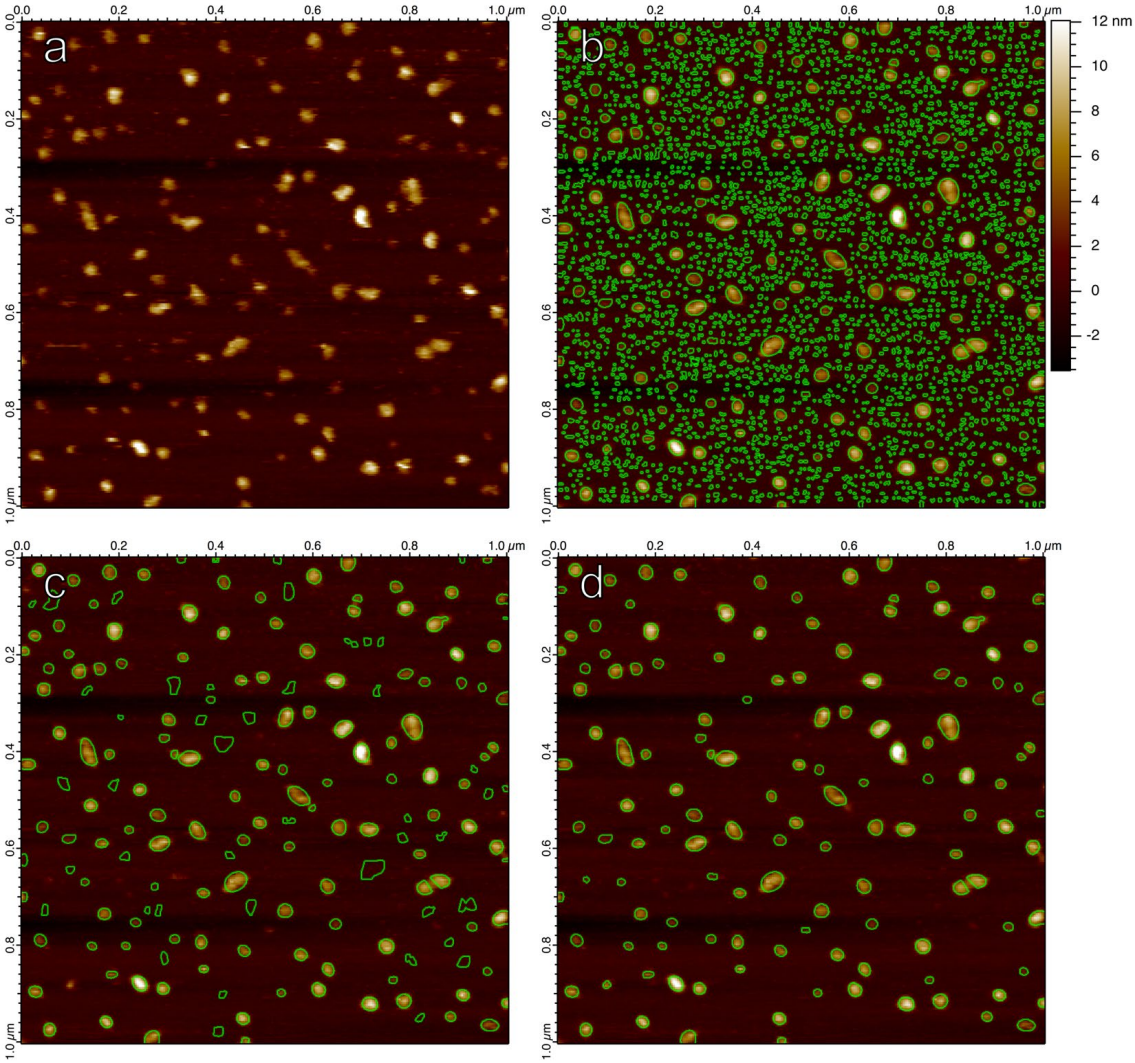
(**a**) An unprocessed AFM image displaying translocase complexes in lipid bilayer, supported on mica^41^, with lateral pixel resolution 3.9 nm. (**b**) All non-overlapping Hessian blobs, extracted from the raw image. Only bright blobs considered. Subpixel boundaries calculated to 1/3 pixel resolution (1.3 nm). (**c**) All Hessian blobs with a scale of (8 nm)^2^ (4 pixel units^2^) or greater, set manually to eliminate most few-pixel blobs. (**d**) All Hessian blobs with blob strength 4.9 · 10^−19^ or greater, set manually to capture visually prominent molecules.

Demonstrated in Fig. 4, thousands of non-overlapping Hessian blobs may exist in a 512 × 512 pixel AFM image, many corresponding to few-pixel, insignificant blobs. Analyzing all Hessian blobs is absolutely valid, though we often wish to consider only a subset of Hessian blobs which likely represent biomolecules of interest. One approach is to set a minimum scale *t*_0_ required for a candidate Hessian blob (Fig. 4c). This method effectively eliminates few-pixel features due to noise and other small-scale structures. However, setting only a minimum scale still allows for larger blobs which are very weak (have a negligible height and show a low blob strength).

Our preferred method of culling Hessian blobs is to consider blobs of any scale, but only allow those with some minimum blob strength (Fig. 4d). Blobs are first discovered as local maxima of the blob detector ${\rm{\det }}\,{ {\mathcal H} }_{norm}L$ – we use the value of ${\rm{\det }}\,{ {\mathcal H} }_{norm}L$ at the scale-space maximum as a natural and simple measure of blob strength. Enforcing a minimum blob strength introduces the only significant degree of freedom present in the algorithm, and it may be set in a number of ways, including automatically. One may set the minimum blob strength by eye to only capture visually prominent molecules within the image. Alternatively and more quantitatively, one may use any number of well-known clustering methods, such as Otsu’s method, balanced histogram thresholding, or k-means, to separate significant and insignificant blobs by their blob strength^37^. It is important to note that enforcing a minimum scale or blob strength does not change the blob boundaries, centers, or shapes; it simply dictates which blobs are considered in the analysis. If no appropriate minimum scale or blob strength can be set, all blobs may be considered.

The Hessian blob algorithm with no minimum blob strength is effectively reduced to a zero-parameter algorithm. Other parameters (how finely the scale is sampled in discrete scale-space, the desired level of subpixel resolution) do effect the precision of the Hessian blobs but do not fundamentally alter particle shapes. By contrast, the threshold algorithm has an irremovable dependence on the height threshold parameter, which cannot simply be set to zero like the blob strength parameter and still produce well-founded results. The watershed algorithm has similar dependencies on an initial smoothing level and minimum pixels parameter (see Supplemental Information). The Hessian blob algorithm is, by this measure, further removed from user interaction and human bias than conventional AFM particle detection methods.

## Particle Detection Algorithm Comparison on AFM Imagery

The Hessian blob algorithm was compared with the conventional threshold and watershed algorithms, measured by how closely the algorithms match manually detected particles. Twenty-three 480 × 480 pixel AFM images of a membrane protein (SecYEG) sample on a glass substrate comprised the data set. SecYEG translocons typically protrude <3 nm above the bilayer^10^, and glass substrates produce characteristically wavy backgrounds and localized defects^11^, instead of the atomically flat background produced by mica substrates. Thus, these images are challenging from an automated analysis perspective, as the biomolecules are similar in height to the amplitude of the background variation.

Images were pre-analyzed manually, labelling each individual SecYEG molecule, with 881 in total. Each algorithm was run repeatedly on every image, where algorithm parameters were swept through their relevant range to optimize performance. This corresponds to the minimum blob strength for the Hessian blob algorithm, the height threshold parameter for the threshold algorithm, and a minimum pixels per particle parameter for the watershed. No preprocessing was performed for the Hessian blob algorithm, while background subtraction was performed for the threshold algorithm and smoothing for the watershed. A score was assigned to an algorithm’s performance on a given image based on the error rate as compared to the labelled particles, and a total error rate was computed as an average over all images. Details regarding particle comparisons, parameter optimization, algorithm implementations, and the fully labelled data set are available in the Supplemental Information.

The full analysis over twenty-three 480 × 480 images was computed on a standard laptop computer (2.7 GHz Intel Core i5 processor, 8GB RAM) in the Igor Pro programming language (full code available upon request). On average, the scale-space representation computed in 5.99 ± 0.30 seconds. Computation of the blob detectors ${\rm{\det }}\,{ {\mathcal H} }_{norm}L$ and ${\nabla }_{norm}^{2}L$, detection of blob centers and boundaries, and removal of overlapping particles took 2.55 ± 0.15 seconds on average. The full Hessian blob algorithm took 8.55 ± 0.38 seconds per image on average when using optimal ${\rm{\det }}\,{ {\mathcal H} }_{norm}L$ thresholds. Real-time performance of scale-space interest point detection has been achieved through hybrid multi-scale representations^38^ which may be implemented in future work towards the real-time detection of Hessian blobs.

**Figure 5:**
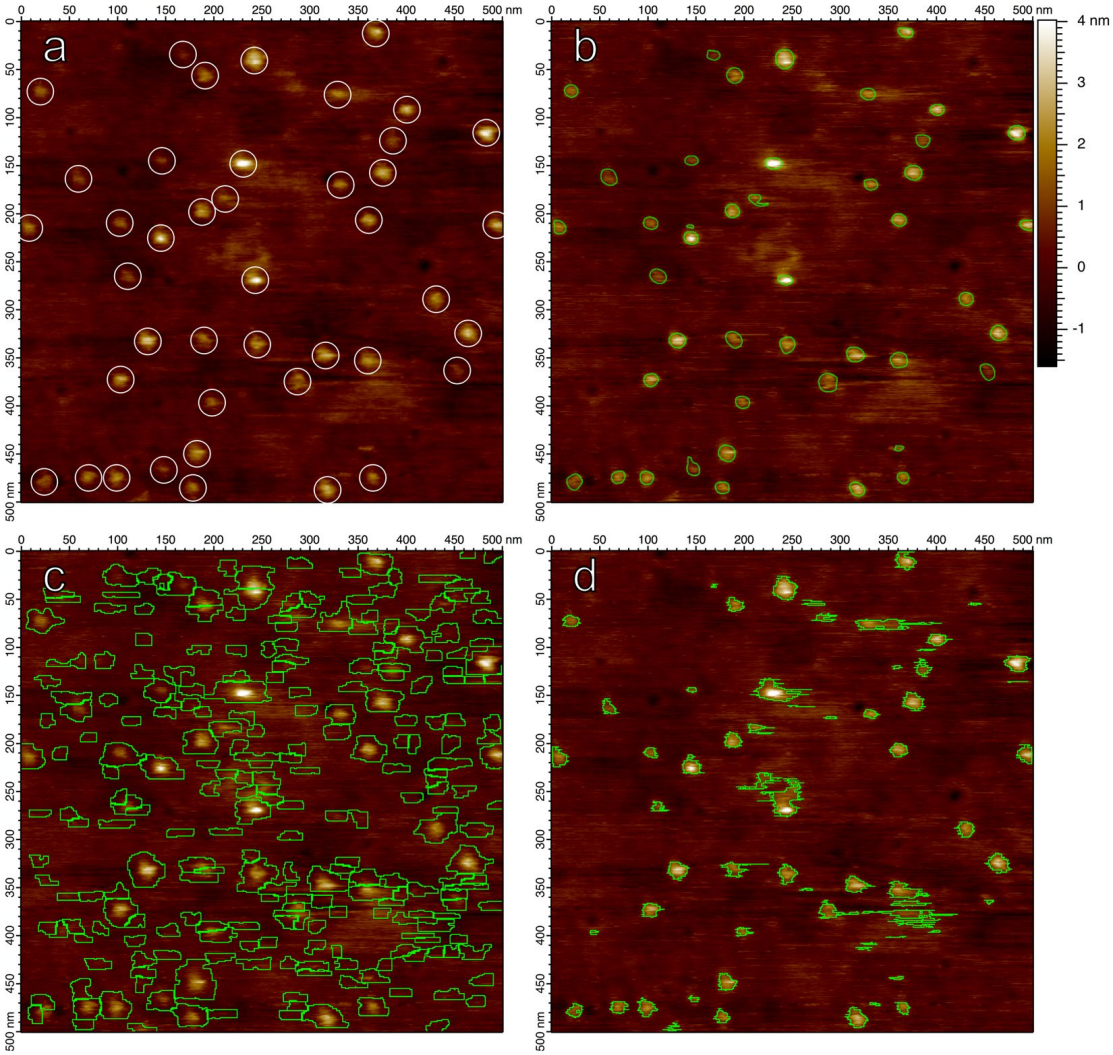
(**a**) The first image in the SecYEG data set, displaying individual translocons in lipid bilayer supported on a glass substrate^11^. Candidate SecYEG molecules are manually labelled with circles of radius 14 nm, regardless of true particle size. (**b**) Particles identified by the Hessian blob algorithm, with the minimum blob strength set such that all candidate particles were identified while minimizing false positive detections. (**c**) Watershed algorithm, with the minimum pixel parameter set in the same manner. (**d**) Height threshold particles, with the height threshold parameter set in the same manner.

The Hessian blob algorithm achieved an average optimal error rate of 5.3% ± 4.9% when checked against manually labelled particles, while the watershed algorithm produced an error rate of 39.6% ± 15.0% and the threshold algorithm produced an error rate of 14.4% ± 8.4%. Figure 5 demonstrates particle boundaries for each algorithm. Figure 5c makes evident that watershed boundaries often expand beyond the particle’s apparent extent, and that boundaries cut directly through particles if there exist multiple maxima within the particle. Figure 5d reveals the give-and-take shortcoming of the threshold algorithm; the appropriate height threshold is not the same for every particle – a compromise must always be struck between not capturing enough of low sitting features, capturing too much background for high sitting features, and picking up noise.

Any algorithm which does not incorporate the concept of scale will be subject to single-pixel scale artifacts. The threshold algorithm and watershed algorithm (Fig. 5c and d) produce an artificial prevalence of perfectly horizontal boundaries for threshold and watershed particles, arising from offsets between line scans, while Hessian blob boundaries remain smooth even without preprocessing.

Another significant advantage of Hessian blobs is that they exist independent of any parameter or user interaction – we simply locate Hessian blobs in scale-space and decide which to consider based on their blob strength. By contrast, threshold particles and boundaries exist only as a function of a user defined height threshold. Watershed particles exist similarly as a function of the initial smoothing level. The threshold and watershed particles indeed change in shape as a function of these parameters, whereas Hessian blobs do not, providing a stable method of defining particles.

**Figure 6:**
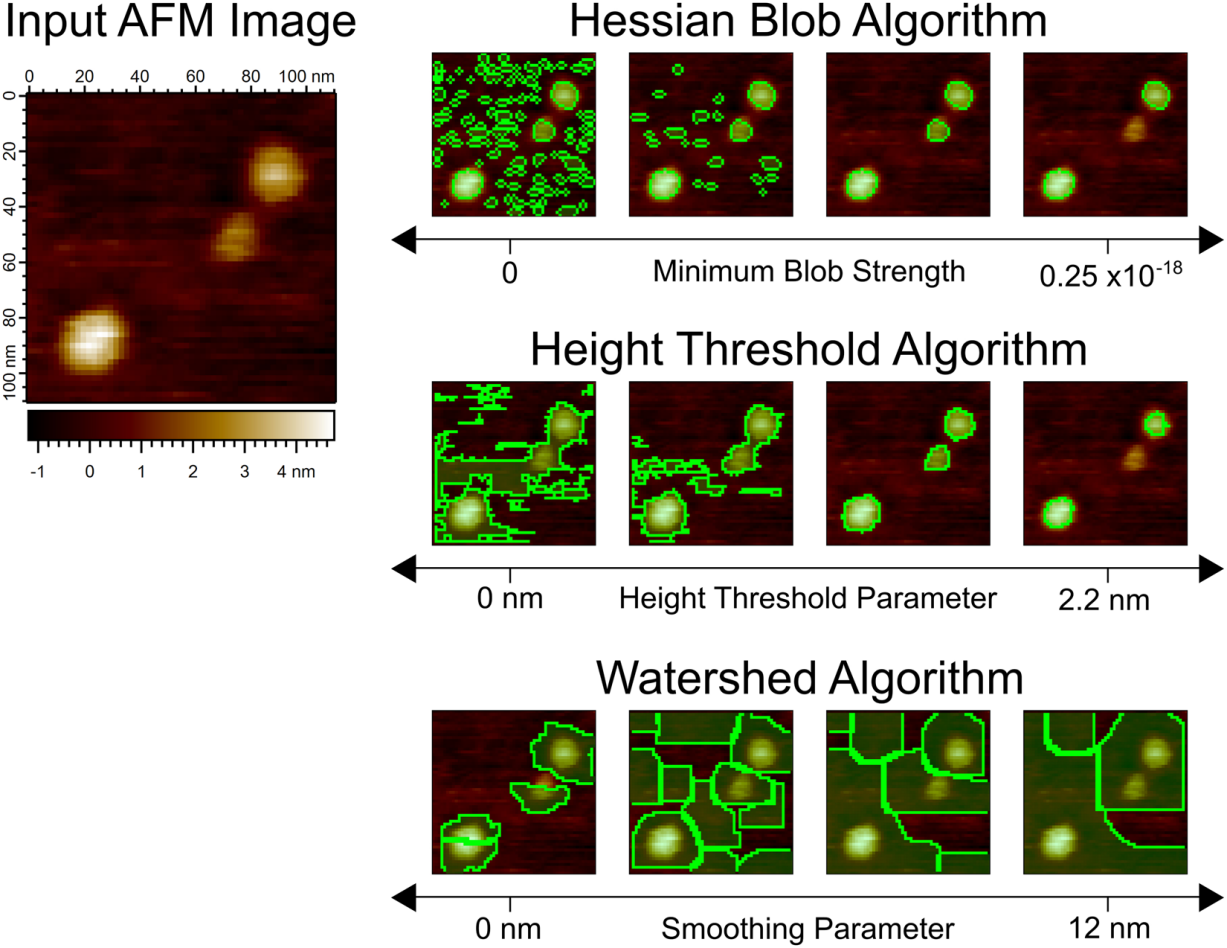
A comparison of particle detection algorithms as user settings are varied, demonstrated on an image of translocase complexes (SecYEG/SecA) on glass. Top row: Hessian blobs computed at single-pixel resolution. The minimum blob strength begins from zero (considering all bright blobs) and increases to the right. Blob boundaries do not deform. Middle row: Threshold algorithm particles, as the height threshold parameter is varied. Particle boundaries deform to include nearby particles and noise at low thresholds. Bottom row: Watershed algorithm particles, as the width *σ* of the discrete Gaussian smoothing kernel is varied, where a 110 pixel minimum was required for every particle. Boundaries expand without limit as the smoothing level increases.

When studying individual biomolecules, a consistent and well-founded method of splitting or joining close particles is necessary for the accurate characterization of the system in terms of average particle area or volume. Figure 6 demonstrates that particles in close proximity may be considered as together or separate for threshold and watershed particles depending on user parameters, whereas this judgement is made independent of the user for Hessian blobs (depending on whether the individual particles or the unit as a whole displays a stronger blob response). Thus, the splitting criteria for Hessian blobs is quantitative and well-founded through measures of blob strength, instead of user set algorithm parameters.

## Hessian Blob Stability in the Presence of Noise and Image Defects

Both components of Hessian blobs, scale-space blob detection and Gaussian curvature region detection, rely upon second-order differential quantities. This implies that Hessian blobs are sensitive only to the local curvature of the image – they are invariant under zero-order (constant) and first-order (planar) perturbations of the image, which occur frequently in atomic force microscopy^36^, and remain resistant even to non-linear defects.

**Figure 7:**
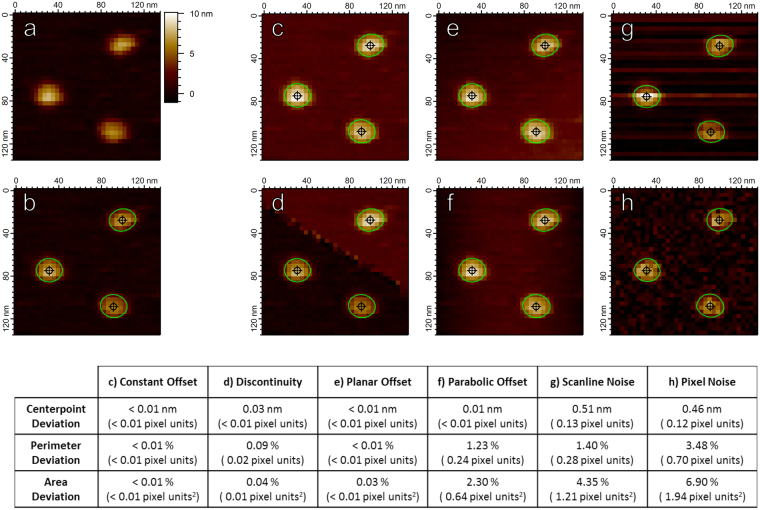
Hessian blobs are stable under image defects commonly seen in AFM images. (**a**) An unprocessed image of translocase complexes on mica with lateral pixel resolution 3.9 nm. (**b**) Hessian blobs discovered in (**a**) with a manually set minimum blob strength 1 × 10^−18^. Blob centers marked by crosshairs, boundaries calculated to 10 times pixel resolution (0.39 nm) and marked by green contours. Panels c-h demonstrate the discovered Hessian blobs after imposing image perturbations. (**c**) Constant Offset: All pixels from (**a**) were given an addition of 2 nm. (**d**) Discontinuity: A fault line was introduced, all pixels above the line were given an addition of 2 nm. (**e**) Tilt: A planar tilt reaching 2 nm over the span of the image was added to (**a**). (**f**) Parabolic: Each horizontal scan line of pixels was given a parabolic addition of up to 2 nm, mimicking the AFM bowing effect. (**g**) Scanline Noise: Each scan line was given a random offset according to a Gaussian distribution of standard deviation 1 nm, mimicking scan line noise. (**h**) Gaussian Noise: Gaussian noise of standard deviation 1 nm was applied to all pixels in (**a**). Table: Average changes in subpixel particle measurements after imposing image perturbations. Center points calculated to subpixel accuracy via second-order Taylor approximation^34^. Particle perimeters and projected areas calculated via the polygon approximation as in the scanning probe microscopy analysis software Gwyddion^5^.

In Fig. 7, the zeroth and first order perturbations (Fig. 7c–e) produce almost no measurable difference in the associated Hessian blobs. The second-order perturbation (Fig. 7f) marginally effect the Hessian blob shape, although the effect is minimal as the parabolic offset provides a large-scale effect (roughly on the scale of the scan size) which minimally perturbs the local curvature of the smaller individual biomolecules. Even after significant degradation of the image by noise (Fig. 7g,h), the Hessian blob characteristics remain recoverable to within a few pixel units. The displayed resistance to noise is a direct result of the scale-space representation, which inherently incorporates scale (and thus smoothing) in the analysis.

## Conclusion

A method for automatic biomolecule detection in AFM imagery was introduced, the Hessian blob algorithm, as an advanced particle detection algorithm which matches the standard of high-precision seen in AFM imagery. A mathematical grounding in scale-space representation theory and differential geometry lends the Hessian blob algorithm a more well-founded definition of a particle which improves on various shortcomings associated with common particle detection methods.

The conventional threshold and watershed algorithms are general image processing methods adopted for use in AFM imagery; these algorithms were shown to be poorly equipped for high-precision, unbiased analyses. Both methods display significant sensitivity to user-set algorithm parameters and demand manual preprocessing of the image. Moreover, threshold and watershed particles are not defined in a manner consistent with the extent of the underlying biomolecule, leading to poorly defined and inconsistent particle shapes.

The Hessian blob algorithm detects particles using well-established blob detection methods and defines boundaries based on local curvature in scale-space, with complete independence from the algorithm parameters. Extension of the image to the scale-space representation makes Hessian blobs resistant to noise, and requires no preprocessing. Hessian blobs, both their centers and boundaries, are also straightforward to extend to subpixel precision. Direct comparison of Hessian blobs against the threshold and watershed algorithm shows higher consistency with manually labelled images. Moreover, the Hessian blob was demonstrated to be a highly resilient structure in the face of common imaging defects related to instrument noise and imperfect imaging conditions.

The framework provided by scale-space representation theory and differential geometry is promising for single molecule biophysics image analysis. Here we present only the first step in a full analysis – precise biomolecule detection. Further work is underway deriving estimates of local background levels below extracted particles, leading to higher precision measurements of common biomolecule metrics such as height and volume. Scale-space signature analysis was also shown to provide insight into substructure elements within detected biomolecules, portending a sophisticated analysis of oligomeric states, protein substructure, and other systems with a nested structure. Verifying the Hessian blob algorithm explicitly against ground truth data is challenging in an AFM context, where factors such as structural dynamics and tip convolution make it difficult to obtain ground truth data. To make progress, one may turn to simulations, where complete knowledge of the system is maintained. In addition to the aforementioned analysis directions, the Hessian blob algorithm may find applications outside of the realm of AFM data analysis.

## Methods

### Membrane protein preparation

SecYEG and SecA were purified from *Escherichia coli* as described^39,40^ and co-assembled into liposomes (*E. coli* polar lipid, Avanti) as described^41^. *Halobacterium salinarum* strain S9 was grown and the purple membrane containing bacteriorhodopsin prepared as described^42^.

### AFM imaging

Images were acquired in tapping mode in fluid at ~30 °C using a commercial instrument (Cypher, Asylum Research Inc.). Details for each sample preparation follow. ***SecYEG and SecYEG/SecA complexes*:** Proteoliposome stock solutions were diluted to 80 nM protein, 80 μM lipid in recording buffer (10 mM HEPES pH 8.0, 200 mM KAc, 5 mM MgAc_2_), immediately deposited on a freshly plasma cleaned glass support^11^ or freshly cleaved mica^10,41^ and incubated for ~20 minutes, followed by rinsing with recording buffer (~300 μl). Biolever mini tips (BL-AC40TS, Olympus) with measured spring constants ~0.06 N/m were used. Spring constants were determined using the thermal noise method. ***Bacteriorhodopsin on mica*:** Following established protocols^43^, equal volumes of stock solution and recording buffer (10 mM Tris pH ~7.6, 150 mM KCl) were mixed before depositing onto a freshly cleaved mica support. After a 1 hr incubation, the sample was rinsed with 300 μl of recording buffer. SNL (Veeco) tips of measured spring constant ~0.4 N/m were used.

## Electronic supplementary material

Supplementary Information
