# Supplementary material for: The Hessian Blob Algorithm: Precise Particle Detection in Atomic Force Microscopy Imagery
Source: Sci Rep. 2018 Jan 17;8:978. doi: 10.1038/s41598-018-19379-x (PMC5772630; doi:10.1038/s41598-018-19379-x)
Supplement: Supplementary file 1 — Supplementary Information [file 41598_2018_19379_MOESM1_ESM.pdf]

Supplementary Information

The Hessian Blob Algorithm: Precise Particle Detection in Atomic Force Microscopy Imagery

Brendan P. Marsh[1,*], Nagaraju Chada[1], Raghavendar Reddy Sanganna Gari[1,], Krishna P. Sigdel[1], and Gavin M. King[a,1,2]**

[1]Department of Physics and Astronomy, and [2]Department of Biochemistry, University of Missouri, Columbia, Missouri 65211, United States of America

[a] *corresponding author*

Computation of The Discrete Scale-Space Representation

Representing an image as a continuous function of two variables $I(x, y) : \mathbb{R}^2 \to \mathbb{R}$, the linear scale-space representation[1,2] $L(x, y; t) : \mathbb{R}^2 \times \mathbb{R}_{>0} \to \mathbb{R}$ consists of a continuous family of images derived from I, indexed by the scale parameter t. By its definition, $L(x, y; t)$ is the solution of the heat equation

$$\partial_t L = \frac{1}{2} \nabla^2 L$$

With initial condition provided by the image, $L(x, y; 0) = I(x, y)$. The scale-space representation can intuitively be thought of, for given scale $t_0 > 0$, as an image derived from I in which all features of scale t_0 or smaller have been blurred out by Gaussian smoothing. Explicitly, the scale-space representation is characterized by the convolution

$$L(x, y; t) = I(x, y) * g(x, y; t)$$

where $g(x, y; t)$: $\mathbb{R}^2 \times \mathbb{R}_+ \to \mathbb{R}$ is the two-dimensional normalized Gaussian kernel

$$g(x, y; t) = \frac{1}{2\pi t} e^{-(x^2+y^2)/2t}$$

A discrete analog to the continuous scale-space representation is required to extend the representation to pixelated images. Two significant aspects of the representation which must be

translated with care are the smoothing operation and discretization of the scale parameter. Moreover, an efficient and fast implementation is dependent on an iterative computation in which each layer of the scale-space representation is computed from the previous layer. Important implementation details are provided here, with full details provided by Lindeberg[3].

A logical choice is to discretize the spatial domain according to the pixel spacing provided by the image; however, discretization of the scale parameter $t \in \mathbb{R}_{>0}$ is less obvious. Natural bounds for the scale are provided by the image: the size of a single pixel provides a clear lower bound, as no feature with structure of a finer scale than the pixel size may be captured in the image, and similarly the full size of the image provides a natural upper bound. Sampling of the scale within this range should be achieved not linearly but geometrically[1] such that $t_{k+1} = r \cdot t_k$ for some constant ratio $r > 1$. We often begin with a minimum scale corresponding to one half of the pixel size and use a ratio $r = 1.5$. The resulting discrete scale-space representation takes the form of a stack of images with increased levels of smoothing.

The Gaussian kernel is the unique smoothing kernel which must be used to achieve the continuous scale-space representation[3]. Its uniqueness stems from fundamental scale-space axioms which should be obeyed by a one-dimensional scale-space kernel, including non-maxima creation (the total number of maxima should not increase after smoothing) and the semi-group property $g(\cdot\,; t_1 + t_2) = g(\cdot\,; t_1) * g(\cdot\,; t_2)$. Thus, irrespective of the type of noise present in the image, the Gaussian kernel should be used such that the axioms of scale-space are satisfied. In the discrete case, although it is tempting to use a sampled Gaussian kernel, it has been shown that these scale-space axioms no longer hold. Thus, the discrete analog of the Gaussian kernel must be used[3,4], which in one-dimension is given by

$$T(n; t) = e^{-t} I_n(t)$$

where $I_n(t)$ is the modified Bessel function of the first kind with integer order n. This kernel indeed obeys the non-maxima creation property and the semi-group property $T(n; t_1 + t_2) = T(n; t_1) * T(n; t_2)$. Separability allows for simple extension to the two-dimensional discrete Gaussian kernel analog $T(n, m; t) = T(n; t) * T(m; t)$. The discrete scale-space representation of a pixelated image may then be accurately characterized by the convolution

$$L(x, y; t) = I(x, y) * T(n, m; t) = \sum_{n=-\infty}^{\infty} \sum_{m=-\infty}^{\infty} T(n, m; t) I(x-n, y-m)$$

The convolution kernel must be truncated to a finite support region. Here the summation limit N is chosen so that the integrated error of the kernel $(1 - \sum_{n=-N}^{N} T(n; t))$ is less than 1%. If necessary, formal bounds on the error associated with truncation have been derived, allowing one to choose the summation limit N to bound the error to any desired level[5].

Smoothing operations by convolution, with smoothing kernels of increasingly large support region in this case, are computationally expensive. Efficient computation of the discrete scale-space representation is dependent on iterative convolution which furthermore makes use of the separability of the discrete Gaussian kernel. For any level of scale, the one-dimensional kernel $T(n; t)$ may be applied in both spatial dimensions more efficiently than applying the two-dimensional kernel $T(n, m; t)$. Then the semi-group property $T(\,\cdot\,; t_1 + t_2) = T(\,\cdot\,; t_1) * T(\,\cdot\,; t_2)$ allows for successive layers of the scale-space representation to be computed from the previous layer using kernels with smaller support regions, resulting in faster execution. In full, the discrete scale-space representation is computed iteratively as follows.

$$L(x, y; t_0) = \sum_{n=-N}^{N} T(n; t_0) \left[\sum_{m=-N}^{N} T(m; t_0) I(x-n, y-m) \right]$$

$$L(x, y; t_{k+1}) = \sum_{n=-N}^{N} T(n; t_{k+1} - t_k) \left[\sum_{m=-N}^{N} T(m; t_{k+1} - t_k) L(x-n, y-m; t_k) \right]$$

Blob Detectors

A general normalized blob detector function $\mathcal{B}_{norm} L(x, y; t)$, such as those considered here and studied by Lindeberg[6], behaves as follows. For a fixed scale t_0, the magnitude of $\mathcal{B}_{norm} L(x, y; t_0)$ attains local extrema on blob centers. This allows for the detection of blobs at any scale by identifying points where $\nabla(\mathcal{B}_{norm} L(x, y; t_0)\,) = 0$, with the gradient operator $\nabla = \partial_x \hat{\imath} + \partial_y \hat{\jmath}$. For a fixed spatial position (x_0, y_0), the response of $\mathcal{B}_{norm} L(x_0, y_0; t)$ as a function of the scale t is known as the scale-space signature. At certain scales, the scale-space signature may attain local extrema such that $\partial_t(\mathcal{B}_{norm} L(x_0, y_0; t)) = 0$. These extremal points are used to

generate hypotheses about natural scales of the image, providing a method of automatic scale-selection. Points in scale-space $(x^*, y^*; t^*)$ which satisfy both conditions, such that $\nabla(\mathcal{B}_{norm}L\,)(x^*, y^*; t^*) = 0$ and $\partial_t(\mathcal{B}_{norm}L)(x^*, y^*; t^*) = 0$, are in general defined as scale-space extrema, and in the context of blob detection identify points in scale-space which correspond to both the spatial center and scale of blobs.

Construction of the blob detectors $\nabla^2_{norm}L$ and $\det\mathcal{H}_{norm}L$ requires computation of the second-order scale-space derivatives L_{xx}, L_{yy}, and L_{xy}, as well as appropriate γ-normalization. Scale-space derivatives, equivalently Gaussian derivatives, may be computed equally well by convolution of the image with Gaussian derivative kernels or by direct differentiation of the scale-space representation[6].

$$L_{x^\alpha y^\beta}(x, y; t) = \left(\partial_{x^\alpha y^\beta}\, g(x, y; t)\right) * I(x, y) = \partial_{x^\alpha y^\beta}\, L(x, y; t) \quad \text{where} \quad \partial_{x^\alpha y^\beta} = \frac{\partial^\alpha}{\partial x^\alpha}\frac{\partial^\beta}{\partial y^\beta}$$

We choose to compute the discrete scale-space representation explicitly and directly apply numerical differentiation. The central difference formulas with error of order $O(h^4)$ were used, as the more common formulas with error term of order $O(h^2)$ do not produce rotationally symmetric results. These central differences may be computed by convolution of the scale-space representation with the derivative kernels below

$$L_{xx}(x, y; t) = \frac{1}{12(\Delta x)^2}\, L(x, y; t) * \begin{pmatrix} -1 & 16 & -30 & 16 & -1 \end{pmatrix}$$

$$L_{yy}(x, y; t) = \frac{1}{12(\Delta y)^2}\, L(x, y; t) * \begin{pmatrix} -1 \\ 16 \\ -30 \\ 16 \\ -1 \end{pmatrix}$$

$$L_{xy}(x, y; t) = \frac{1}{144\Delta x\Delta y}\, \left(L(x, y; t) * \begin{pmatrix} -1 & 8 & 0 & -8 & 1 \end{pmatrix}\right) * \begin{pmatrix} 1 \\ -8 \\ 0 \\ 8 \\ -1 \end{pmatrix}$$

where Δx is the pixel spacing in the x direction and Δy the pixel spacing in the y direction. These convolution formulas were used to compute L_{xx}, L_{yy}, and L_{xy} at every point in discrete scale-space.

In general, the amplitude of the derivative response will decrease with increasing scale and smoothing. To preserve the response of scale-space derivatives across scale, the γ-normalized derivative operator $\partial_{\xi,\gamma-norm} = t^{\gamma/2}\partial_x$ corresponding to the change of variable $\xi = x/t^{\gamma/2}$ is used[6]. Noting that the choice of $\gamma = 1$ produces a dimensional variable ξ, we use $\gamma = 1$ to achieve a perfect scale invariant response[7]. This implies that if two functions $f(x)$ and $g(x)$ are equivalent up to a scaling transformation, $f(x) = g(\alpha x)$ for $\alpha \in \mathbb{R}$, that their responses under a $\gamma = 1$ normalized derivative operator D_{norm} will be related by $D_{norm}f(x;t) = D_{norm}g(\alpha x; \alpha^2 t)$. In particular, suppose a scale-space extremum of magnitude A is discovered for $D_{norm}f$ at the point (x_0, t_0). Then $D_{norm}f(x_0; t_0) = A$. For a scale invariant D_{norm}, a scale-space extremum of $D_{norm}g$ will be found at the point $(\alpha x_0, \alpha^2 t_0)$ also with magnitude A, such that $D_{norm}g(\alpha x_0; \alpha^2 t_0) = A$. Thus, the magnitude of scale-space extrema are conserved, allowing for direct comparison of image features of any scale.

The normalized Laplacian of Gaussian $\nabla^2_{norm}L$ and determinant of the Hessian $\det \mathcal{H}_{norm}L$ blob detector functions are then computed directly from the numerical derivatives, and by applying $\gamma = 1$ normalization.

$$\nabla^2_{norm}L(x,y;t) = t(\, L_{xx}(x,y;t) + L_{yy}(x,y;t)\,)$$

$$\det \mathcal{H}_{norm}L\,(x,y;t) = t^2\big(\, L_{xx}(x,y;t)L_{yy}(x,y;t) - L^2_{xy}(x,y;t)\,\big)$$

The Hessian Blob Algorithm

The workflow of the Hessian blob algorithm is outlined here sequentially.

1. **Computation of Scale-Space Representation:** From an input image $I(x,y)$, the scale-space representation $L(x,y;t)$ is computed as outlined in the first section of the Supplementary Information and stored in memory. Although it possible to directly compute the blob detectors via convolution with Gaussian derivative kernels, we choose to precompute the scale-space representation separately and apply numerical differentiation to compute the blob detectors, as it is more efficient when computing multiple blob detectors.

2. **Computation of Blob Detectors:** The blob detectors $\det \mathcal{H}_{norm}L$ and $\nabla^2_{norm}L$ are then computed directly from the scale-space representation $L(x, y; t)$ as outlined in the preceding section of the Supplementary Information and also stored in memory.

3. **Detection of Hessian Blob Seeds:** Seeds of Hessian blobs in scale-space are then discovered by searching for local scale-space maxima of $\det \mathcal{H}_{norm}L$ over all non-boundary pixels. Scale-space maximal points must be maximal in both space and scale, this requires checking all 26 direct neighbors in three-dimensions. Although, in practice most points will be disqualified as scale-space maxima after comparison with less than all 26 neighbors, thus the search does not require 26 checks for every pixel in scale-space. Moreover, we enforce a minimum blob strength (defined as the value of $\det \mathcal{H}_{norm}L$ at the scale-space maximum) to prune insignificant blobs. Thus, any pixel which does not meet the minimum $\det \mathcal{H}_{norm}L$ response does not need to be checked against any of its neighbors.

4. **Validation of Blob Seeds:** After a candidate Hessian blob seed $(x_0, y_0; t_0)$ is discovered which demonstrates a strong enough blob response, the sign of the blob (bright blob or dark blob) is recovered via the sign of $\nabla^2_{norm}L(x_0, y_0; t_0)$. The type of blob may optionally be restricted to bright or dark blobs by enforcing a negative (bright blob) or positive (dark blob) $\nabla^2_{norm}L$ at the scale-space maximum point $(x_0, y_0; t_0)$. Here we restrict to only bright blobs which may correspond to biomolecules protruding above the lipid bilayer; however, dark blobs may be of interest in the study of membrane pores or protein channels through the bilayer. Finally, we further enforce the criteria introduced in the SIFT algorithm[8] which sets a limit on the ratio of the principal curvatures at the scale-space maximum to eliminate spurious responses of $\det \mathcal{H}_{norm}L$ along edges. As recommended by the authors, we set a maximum principal curvature ratio of 10. The principal curvatures are not computed directly, as it is shown by the authors that the ratio of the principle curvatures r can be related to the bump detectors $\det \mathcal{H}_{norm}L$ and $\nabla^2_{norm}L$. For any candidate scale-space maximum $(x_0, y_0; t_0)$, we enforce the following criterion.

$$\frac{(\nabla^2_{norm}L(x_0, y_0; t_0))^2}{\det \mathcal{H}_{norm}L(x_0, y_0; t_0)} = \frac{(r+1)^2}{r} < \frac{(10+1)^2}{10} = 12.1$$

5. **Hessian Blobs Boundary Detection:** A Hessian blob seed in scale-space $(x_0, y_0; t_0)$ which meets the above criteria is then filled out to localize the boundary of the blob. The blob's extent is the set of connected pixels around the scale-space maximum, isolated at the scale t_0, for which the Gaussian curvature remains positive. Direct computation of the Gaussian curvature is not required either, as the sign of the Gaussian curvature and the sign of $\det \mathcal{H}_{norm} L$ are equivalent. Thus, regions of positive Gaussian curvature are equivalent to regions of positive $\det \mathcal{H}_{norm} L$. We use the scanline fill algorithm, popular in common image editing software, to efficiently determine the region of positive of $\det \mathcal{H}_{norm} L$ around the point (x_0, y_0) at the scale t_0, providing the region associated with the Hessian blob.

6. **Projection Overlap Algorithm:** Depending on the context of the analysis, it may be important that detected particles do not overlap. While Hessian blobs do not overlap in three-dimensional scale-space, it is possible and common for them to overlap when projected back down to the two-dimensional spatial domain. Any number of solutions are possible for eliminating overlapping blobs, and the appropriate solution will depend on the context of the analysis. Here we use the simple solution which gives priority to blobs of higher strength, defined as the value of $\det \mathcal{H}_{norm} L$ at the blob center (the scale-space maximum). Computationally, a table containing the strengths of all Hessian blobs is generated and then sorted from highest to lowest strength. The blob of highest strength is then projected out of scale-space to the spatial domain. The next strongest blob is then considered; if it overlaps with any blob already projected to the spatial domain, it is discarded, otherwise it is projected to the spatial domain as well. The previous step is repeated until all blobs have been projected or discarded.

7. **Subpixel Refinement of Center Points:** Subpixel refinement to the blob center may be implemented by approximating the subpixel position of the scale-space maximum to second order. Following the implementation of Brown and Lowe[9], the second-order Taylor expansion of $\det \mathcal{H}_{norm} L$ is computed in the neighborhood around the (pixel resolution) scale-space maximum $\boldsymbol{x_{pix}} = \left(x_{pix}, y_{pix}; t_{pix}\right)^{\mathrm{T}}$, where T denotes the transpose. Central difference formulas with error term of order $O(h^4)$ are used to compute the first and second order

derivatives of $\det \mathcal{H}_{norm} L$ at $\boldsymbol{x_{pix}}$. The refinement of the scale-space maximum location is then computed by setting the derivative of the Taylor expansion to zero, producing the following formula for the sub-pixel resolution scale-space maximum.

$$\boldsymbol{x_{subpix}} = \boldsymbol{x_{pix}} - \left(\frac{\partial^2}{\partial \boldsymbol{x}^2} \det \mathcal{H}_{norm} L\right)^{-1} \cdot \left(\frac{\partial}{\partial \boldsymbol{x}} \det \mathcal{H}_{norm} L\right)$$

8. **Subpixel Refinement of Boundaries:** Subpixel refinement of the Hessian blob boundary is achieved by interpolating $\det \mathcal{H}_{norm} L$ to any desired resolution before determining the region of positive $\det \mathcal{H}_{norm} L$ around the scale-space maximum. Two common interpolation methods, bicubic and bilinear, are often used in image editing software and in scientific contexts while many other methods have been proposed[10]. We note that bicubic interpolation preserves continuous derivatives after interpolation, producing smoother Hessian blob boundaries, at the expense of higher computational load. To further save unnecessary computations, $\det \mathcal{H}_{norm} L$ is only interpolated to higher resolution within a one pixel window of the original (pixel resolution) boundary, as the interpolated boundary must fall within that window. After interpolation, the scanline fill algorithm is again used to determine the region of positive $\det \mathcal{H}_{norm} L$ around the associated scale-space maximum, now to subpixel precision.

Traditional Algorithm Implementations

The traditional height threshold algorithm was implemented in standard form for AFM analysis. The raw image was first subject to a preprocessing step involving a second order flattening to remove the "bowing effect" common in AFM images[11]. A user defined mask is set to eliminate non-background particles from the flattening, then the second order polynomial of best fit (by least squares) was subtracted from each line scan. The height threshold parameter is then used to threshold the processed image, resulting in a binary mask marking pixels above and below the threshold. Connected sets of pixels above the threshold, which contain at least 20 pixels in total (determined qualitatively to eliminate insignificant small-scale particles and noise), are then labelled as particles.

The classical watershed algorithm was implemented via the optimal minimum spanning forest implementation[12] to segment the image into catchment basins – intuitively thought of as regions for which drops of water placed at any pixel in the region "drain" (by steepest descent) to the same minimum. To detect peaks instead of valleys, the image is first inverted. To minimize the high number of local minima in the image, which will each produce their own catchment basin, discrete Gaussian smoothing was applied to the image before running the algorithm. The amount of smoothing, dictated by the width of the Gaussian smoothing kernel, is set by the user as an algorithm parameter. After manual optimization, we held the smoothing level constant at 1 pixel unit width for the algorithm comparison on the SecYEG data set. The watershed algorithm was then executed, resulting in a full segmentation of the image. To minimize the number of regions which do not contain particles of interest, a minimum pixels per region criterion is introduced as another algorithm parameter, eliminating regions which do not contain the minimum number of required pixels.

Algorithm Comparison Metrics and Labelled SecYEG Data Set

Particles were manually detected in the SecYEG data set and labelled by a single pixel located in the center of the biomolecule. After particles were identified by an algorithm, they were compared to the labels. For any particle identified by the algorithm, hereon regarded as an algorithm particle, it was checked to see if the algorithm particle coincided with a label. If it did not, it was categorized as a type I error. If the algorithm particle indeed coincided with a label, the algorithm particle was labelled as a correct particle. After all algorithm particles were considered, any remaining undiscovered labels missed by the algorithm were considered as type II errors. The error rate for the i'th image Error_i and the total error rate for an algorithm Error_{Total} was calculated as below, where N_i is the number of labelled particles in the i'th image and N_{Total} is the total number of labelled particles in all images.

$$\mathrm{Error}_i = \frac{\text{Type I Errors} + \text{Type II Errors}}{\text{Correct Particles}} \quad , \qquad \mathrm{Error}_{Total} = \frac{1}{N_{Total}} \sum_{i=1}^{N} \mathrm{Error}_i * N_i$$

Each algorithm was executed repeatedly on every image, where parameters were swept through their relevant range to optimize performance. For the Hessian blob algorithm, the

minimum blob strength (det $\mathcal{H}_{norm}L$ at the scale-space maximum) varied from zero to the strength of the strongest blob. For the threshold algorithm, the height threshold parameter used to construct a binary image was varied between the image minimum and maximum. A minimum of 20 total pixels was also enforced for the threshold algorithm to minimize spurious particles arising from noise. For the watershed algorithm, the minimum pixels per particle parameter was varied from 0 to size of the largest particle.

The Hessian blob algorithm was run without preprocessing of the raw images. Conversely, the threshold and watershed algorithms required preprocessing for effective particle detection. A second order flattening was applied to each scan-line of the image before running the threshold algorithm to normalize the background level, although the waviness of the glass substrate eliminates the possibility of fully removing the background variation. Gaussian smoothing of width $\sigma = 1$ pixel unit was applied to the image before applying the watershed algorithm to minimize spurious maxima.

The full set of twenty-three SecYEG images are given below. Every particle label is represented by a circle of radius 14nm around the labelled pixel, irrespective of the actual size of the biomolecule.

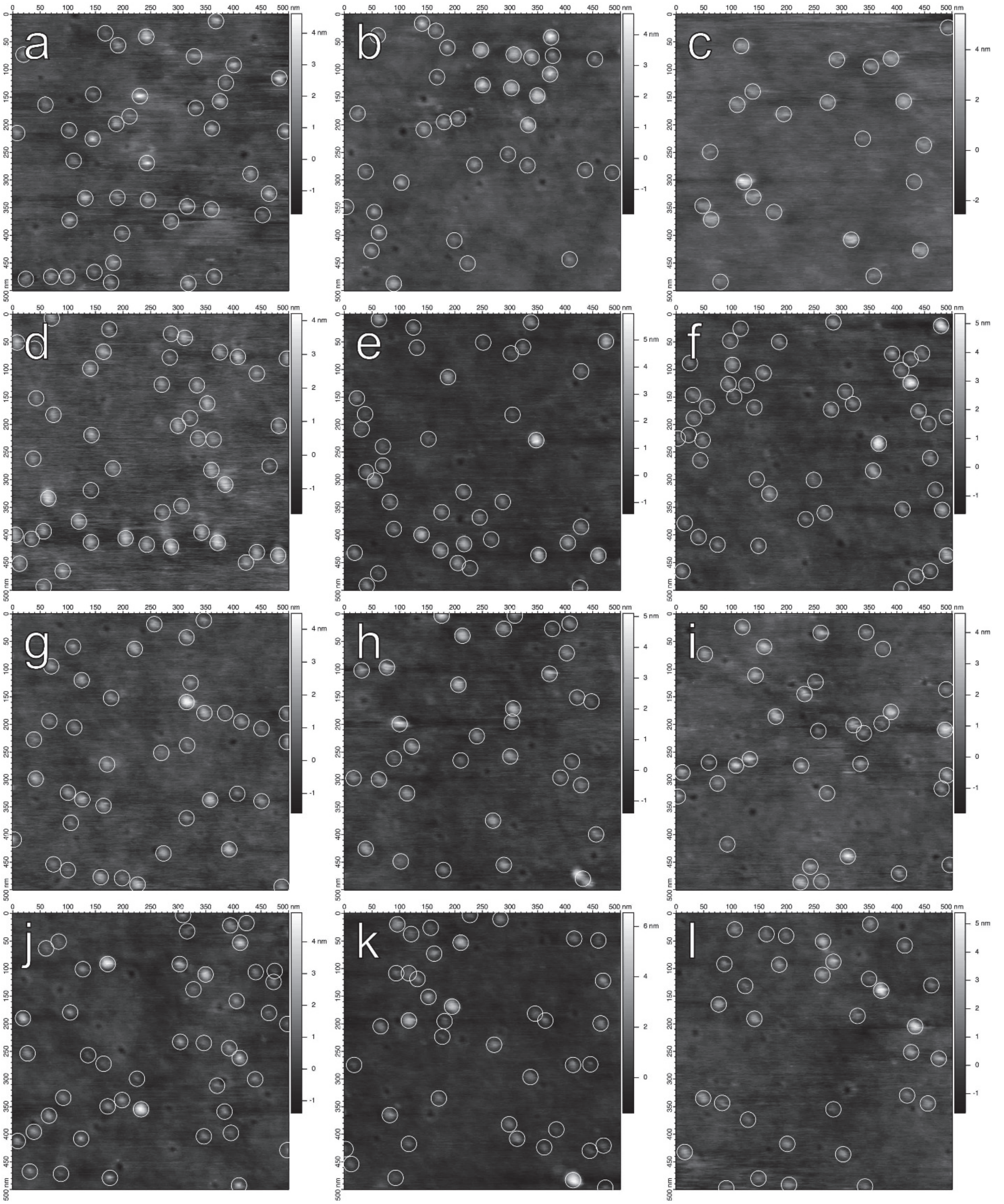
a
b
c
d
e
f
g
h
i
j
k
l

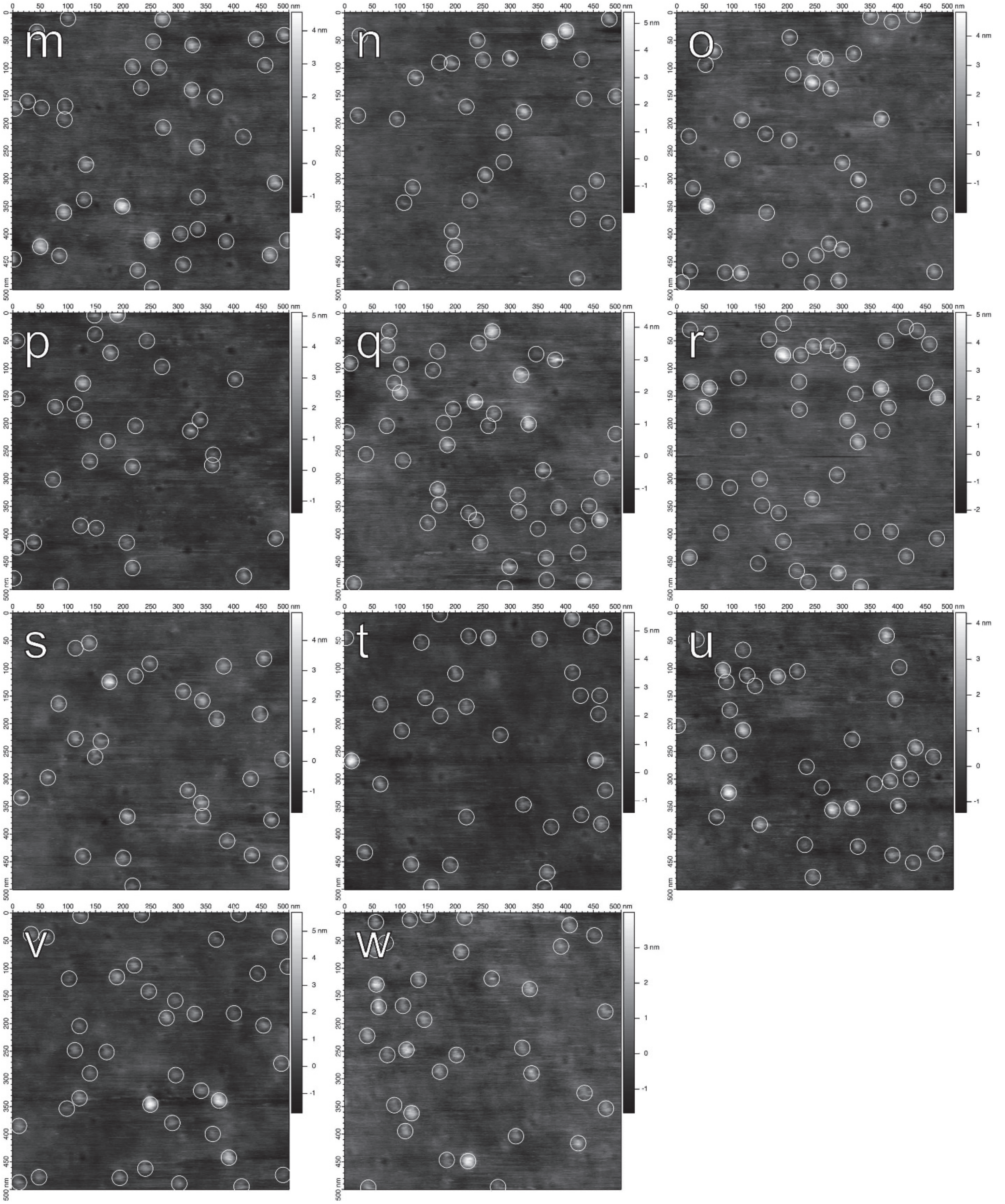
m
n
o
p
q
r
s
t
u
v
w
0 50 100 150 200 250 300 350 400 450 500 nm

References

1 Koenderink, J. J. The structure of images. *Biological Cybernetics* **50**, 363-370, doi:10.1007/BF00336961 (1984).

2 Lindeberg, T. *Discrete Scale-Space Theory and the Scale-Space Primal Sketch* Ph.D. thesis, Royal Institute of Technology, (1991).

3 Lindeberg, T. Scale-space for discrete signals. *IEEE Transactions on Pattern Analysis and Machine Intelligence* **12**, 234-254, doi:10.1109/34.49051 (1990).

4 Norman, E. A Discrete Analogue of the Weierstrass Transform. *Proceedings of the American Mathematical Society* **11**, 596-604, doi:10.2307/2034718 (1960).

5 Lindeberg, T. On the Construction of a Scale-Space for Discrete Images. 51 (KTH Royal Institute of Technology, 1988).

6 Lindeberg, T. Feature Detection with Automatic Scale Selection. *Int. J. Comput. Vision* **30**, 79-116, doi:10.1023/a:1008045108935 (1998).

7 Florack, L. M. J., ter Haar Romeny, B. M., Koenderink, J. J. & Viergever, M. A. Scale and the differential structure of images. *Image and Vision Computing* **10**, 376-388, doi:http://dx.doi.org/10.1016/0262-8856(92)90024-W (1992).

8 Lowe, D. G. Distinctive Image Features from Scale-Invariant Keypoints. *International Journal of Computer Vision* **60**, 91-110, doi:10.1023/B:VISI.0000029664.99615.94 (2004).

9 Brown, M. & Lowe, D. Invariant Features from Interest Point Groups. *Proceedings of the British Machine Vision Conference*, 253-262 (2002).

10 Li, X. & Orchard, M. T. New edge-directed interpolation. *Trans. Img. Proc.* **10**, 1521-1527, doi:10.1109/83.951537 (2001).

11 Tsaftaris, S. A., Zujovic, J. & Katsaggelos, A. K. in *2008 16th European Signal Processing Conference.* 1-5.

12 Cousty, J., Bertrand, G., Najman, L. & Couprie, M. *Watersheds, minimum spanning forests, and the drop of water principle*, https://hal.inria.fr/hal-01113462 (2007).
